# Optical coherence tomography angiography (OCTA) findings in Serpiginous Choroiditis

**DOI:** 10.1186/s12886-020-01527-5

**Published:** 2020-06-30

**Authors:** Sergio Macedo, Dominika Pohlmann, Matthias Lenglinger, Uwe Pleyer, Antonia M. Joussen, Sibylle Winterhalter

**Affiliations:** Department of Ophthalmology, Campus Virchow- Klinikum, Charité – University Medicine Berlin, corporate member of Freie Universität Berlin, Humboldt- Universität zu Berlin and Berlin Institute of Health, Augustenburger Platz 1, 13353 Berlin, Germany

**Keywords:** Fluorescein angiography, Fundus autofluorescence, Indocyanin green angiography, Optical coherence tomography angiography, Posterior uveitis, Serpiginous Choroiditis

## Abstract

**Background:**

To describe changes in the retina/choroid in patients with Serpiginous Choroiditis (SC) by Optical Coherence Tomography Angiography (OCTA) in a multimodal imaging approach.

**Methods:**

Prospective, monocentric study of 24 eyes of 12 consenting patients diagnosed with SC, who underwent OCTA, which was analyzed and compared to other methods such as enhanced depth imaging-OCT, fluorescein angiography, indocyanine green angiography, and fundus autofluorescence.

**Results:**

The study group consisted of 9 patients with peripapillary SC, 1 macular SC, and 2 atypical cases. All eyes presented an inactive SC confirmed by standard imaging. OCTA demonstrated the lesions tridimensionally in great detail. There was no difference in the angioarchitecture among the 3 forms of SC. A loss of the choriocapillaris/retinal pigment epithelium left a “window-defect”, where the vessels of larger caliber of the choroid became recognizable and their appearance inverted (“white-on-black”). A relationship between the presence of segmentation errors (SE) in the slabs and low visual acuity was established with a one-way ANOVA.

**Conclusions:**

OCTA was able to non-invasively assess vascular lesions of the choroid/retina in patients with SC with a high degree of correlation to other diagnostic modalities. Consequent long-term assessments could lead to a better understanding of disease progression.

## Background

Serpiginous choroiditis (SC) is a recurrent inflammatory primary choriocapillaropathy, belonging to a group of rare idiopathic diseases called “white-dot syndromes”, which were first described by Ezra in 1995 [1]. The disease commonly occurs bilaterally and asymmetrically, progressing in a centrifugal and phased manner. Patients, mostly between the age of 20 and 60 years, usually complain of blurry vision, scotomas, and metamorphopsia, equating to a diminished quality of life with irreversible loss of vision in cases where the macula is affected [2, 3].

Three types of SC are currently recognized depending on the primary location of inflammation [4, 5]. The “peripapillary form” accounts for approximately 80% of cases and begins near the optic disc with a late affection of the macular region, whereas the “macular form” has a poor initial visual prognosis because of the early disease manifestation of the macula. In contrast to this, the “atypical form” starts in the periphery in a partly multifocal manner, may reach the macular region and possibly initiates as acute posterior multifocal placoid pigment epitheliopathy [6]. Before the diagnosis of SC can be determined, tuberculosis related uveitis has to be excluded [7].

There is currently no consensus regarding its optimal treatment options, which consists mostly of immunosuppressive substances and biologicals [8, 9]. However, disease recurrence is often observed despite therapy. A better anticipation of drug response and disease progression could help lower treatment burden.

In the past years, a multimodal imaging approach has been described to facilitate diagnosis and to assess disease progression and re-activation in white-dot syndromes [10].

The gold standard for retinal imaging is fluorescein angiography (FA) [11].

However, indocyanine green angiography (ICGA) is probably the current best tool to indicate the true extent of choriocapillaropathies [12–14].

But all current imaging modalities concentrate on the retina, retinal pigment epithelium (RPE), or choroid. However, optical coherence tomography angiography (OCTA) is a new 3D non-invasive diagnostic method which is capable of examining the retinal and choroidal vasculature concurrently and allows for a detailed study of vascular perfusion in all layers via motion contrast imaging.

Until now, there are only case reports concerning OCTA and one case series encompassing 3 patients in SC [15]. Therefore, the aim of this study is to describe these findings in greater extent and compare them to known diagnostic tools, such as FA, ICGA, fundus autofluorescence (FAF), and enhanced depth imaging optical coherence tomography (EDI-OCT) [16–23].

## Methods

### Patients

This prospective study was approved by the local ethics committee (EA4/055/16) and adhered to the tenets of the declaration of Helsinki. Patients with SC were included between April and November 2016 after informed consent was given, so that their data could be used in this study as well as for the ability to perform a clinical examination and imaging. Diagnosis of SC was confirmed after exclusion of infectious diseases such as tuberculous uveitis [7].

### Clinical examination and imaging

Full ophthalmologic examination, including snellen visual acuity test, Goldmann contact tonometry, slit-lamp examination, and fundoscopy with a 90-diopter lens, was performed and all patients received the following imaging modalities: EDI-OCT, FA, ICGA, and FAF. Visual acuity results were converted into a logMAR scale, according to the visual acuity measurement standard from the international council of ophthalmology.

All imaging modalities were carried out by an experienced ophthalmic photographer with the Heidelberg SPECTRALIS OCT, Heidelberg Engineering, Heidelberg, Germany (v1.9.2014.0 - Acquisition Module v6.4). The technical aspects of the machine were standardized as follows: for the EDI-OCT - Scan angle of 20°, 512 A-Scans for 19 Sections, inter-section distance of 250 μm, scaling of 11.5 μm/pixel and an ART of 100 frames; for the FAF - Scan angle of 55°, 768 A-Scans, scaling of 20 μm/pixel, and an ART of 100 frames.

FA and ICGA were performed. The early, intermediate and late phases of both imaging modalities were examined and the activity state was determined.

Inactive SC was identified based on clinical findings of fundoscopy and on multimodal imaging.

Active SC can lead to worsening of the vision, metamorphopsia, inflammation signs at the border of the previous lesions and neovascularization at the borders of the lesion. In FAF, active SC is described by images showing a peripheral hypoautofluorescence area surrounding the hyperautofluorescent borders of the lesions. Old atrophic areas are (because of the loss of choriocapillaris) completely hypoautofluorescent [24–26]. In FA, active SC shows hypofluorescence surrounded by a hyperfluorescent border, with staining and leakage in the late phases. Inactive SC lesions are hypofluorescent at first, acquiring a hyperfluorescent edge later. ICGA shows hypofluorescent lesions throughout the entire examination, but usually of greater (and more reliable) extent in comparison to FA [27, 28].

### OCT angiography

OCTA images were acquired using a prototype SPECTRALIS OCT device (SPECTRALIS®, Heidelberg Engineering, Heidelberg, Germany) using a prototype software (6.4.204.0) that applied an OCTA acquisition algorithm on which the now commercially released version is based. Images were acquired with an A-scan rate of 70,000 per second and a 15°× 10° scan angle protocol was used. A total of 261 B-scans resulting in images with an axial resolution of approximately 4 μm, within B-scan resolution of approximately 11 μm (6.99μmpixel), and between B-scan resolution of also approximately 11 μm. The OCTA C-scan derived from the B-scans allows for 3D visualization of the different retinal and choroidal vascular plexuses. The scanning frame dimensions were 4.2 × 2.8 mm, being centered at the macula.

A standardized reproduction of the superficial capillary plexus (SCP – at 30 μm ± 30 μm below the inner limiting membrane, ILM, representing the ganglion cell layer), deep capillary plexus (DCP - at 130 μm ± 12.5 μm below the ILM, representing the inner nuclear layer), RPE, Choriocapillaris (at 10 μm ± 0 μm below the Basement Membrane, BM), Sattler’s Layer (at 70 μm ± 10 μm below the BM,) and Haller’s Layer (at 140 μm ± 10 μm below the BM) were attempted based on the “Atlas OCT Angiography in AMD” [29]. Each OCTA produced by the software’s algorithm was analyzed for the presence of the expected anatomy to be found at the respective depth of the retina, this being also based on the “Atlas OCT Angiography in AMD” [29]. For this comparison, interobserver correlation was assessed. The identification and differentiation between the Sattler’s and Haller’s layer were done with recourse to the following criteria: the Sattler’s layer was identified by vessel-like entities in a hyper-intense grayish background, appearing below the choriocapillaris until reaching the Haller’s layer, an area of hypo- and hyper-intense signals corresponding to bigger vessels.

Furthermore, the images were then evaluated with respect to the presence of segmentation errors, which were defined by clear deviations of the segmentation line from the observable path of the anatomical structures, due to imprecise or altogether lacking identification of the true reference point. Afterwards, the images were optimized to improve the analysis. Segmentation boundaries were manually changed to best reproduce the expected anatomy (mostly in the retinal pigment epithelium and below).

### Characterization of lesions

The atrophic area at the level of the choriocapillaris and RPE were measured manually (in mm^2^) after proper and individual segmentation by ML and SM of the corresponding OCTA slab. This was achieved using the linear caliper tool of the OCTA embedded in the Heidelberg Eye Explorer Software (Viewing Module 6.6.0.1), in order to compare both measurements and reliability of the technology across observers.

### Statistics

Statistical Analysis was performed using IBM SPSS Statistics v23. First, we analyzed the data with descriptive and frequency statistics. A t-Test-Paired Two-Sample Test assuming Equal Variances was employed to compare the area of atrophy at the choriocapillaris and RPE of each patient. A Mann-Whitney U Test was used to compare the best corrected visual acuity (BCVA) of patients with SE and patients without SE. A one-way ANOVA-Test was used to assess the relationship between the BCVA and the presence of SE on the OCTA’s. Interobserver agreement for qualitative data was studied using Cohen’s kappa and Lin’s concordance correlation coefficient for quantitative data. The results were regarded as statistically significant if *p* was below 0.05 [30].

## Results

### Patients

Twenty-four eyes of 12 patients with SC were examined at our department of Ophthalmology. Eight patients (67%) were female and 4 male (33%). The mean age of all patients on the date of data collection was 60 +/− 15.6 standard deviation (SD) years.

One patient (2 eyes) was diagnosed with macular SC, 2 with atypical SC, and 9 with peripapillary SC. One eye was excluded due to the presence of significant macular edema and one due to the interruption of the examination, as requested by the patient owing to lightheadedness.

In total, 22 eyes of 12 patients with the diagnosis of currently inactive SC were examined and evaluated. Patient demographics and disease status are shown in more detail in Table 1.

**Table 1:** Patients demographics

Age, years	Sex	Type of Disease	Visual Acuity OD	Visual Acuity OS	Activity OU	Years since first Visit	Years since last recurrence	Treatment OU
81	M	A	20/32	–	Inactive	8	3	Prednisolon
57	W	A	20/400	20/60	Inactive	2.5	2.5	Methotrexate
47	W	M	20/125	20/2000	Inactive	7	7	Methotrexate
34	W	P	20/20	20/20	Inactive	2.8	2.8	Adalimumab
63	W	P	20/25	–	Inactive	15.7	15.7	Azathioprine
73	M	P	20/200	20/100	Inactive	9	2	Ciclosporine
34	W	P	20/20	20/50	Inactive	1.4	1.4	Ciclosporine
68	W	P	20/25	20/25	Inactive	3.7	3.7	Ciclosporine
54	W	P	20/20	20/20000	Inactive	6	3	Interferon-α
68	M	P	20/20	20/40	Inactive	5.8	5.8	Interferon-α
62	M	P	20/2000	20/32	Inactive	1.6	1.6	Methotrexate + Prednisolon
79	W	P	20/20000	20/20	Inactive	6	6	–

*A* atypical; *M* macular; *P* peripapillary; *OD* right eye; *OS* left eye; *OU* both eyes

The mean BCVA was 20/100 [0.71 logMAR (SD 0.96)] with a median of 20/36 [0.25 logMAR (range 0–3)]. Three patients (25%) were under therapy with Cyclosporine A with a daily dose range of 1–3 mg/kg. Two patients (17%) were treated with Interferon alpha-2a (3 Mio. IE every 3 days) and another 2 patients (17%) with Methotrexate (15 mg/week). Other therapies consisted of Azathioprine (75 mg/day), Adalimumab (40 mg s.c. every 2 weeks) in combination with corticosteroids (7.5 mg/day), and Methotrexate (7.5 mg/week) in combination with corticosteroids (7.5 mg/day), each in one patient. One patient did not receive any therapy as shown in Table 1. Mean duration of disease was 5.8 years (SD 4.0) with a median of 5.9 years. Mean time since last recurrence was 4.5 years (SD 4.0) with a median of 3 years.

### Multimodal imaging

**Fig. 1:**
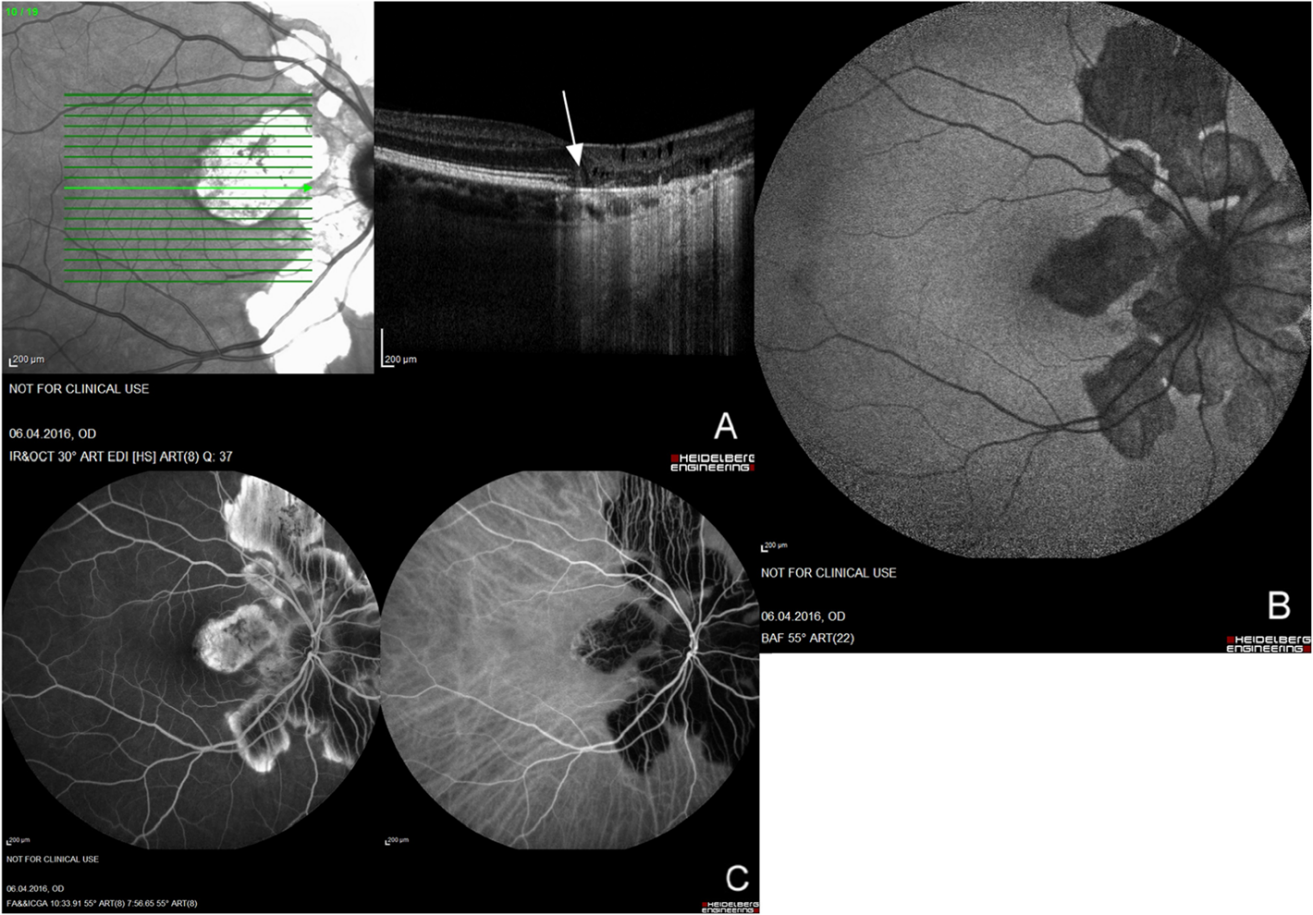
Multimodal imaging of a 53-year-old female patient with peripapillary Serpiginous Choroiditis, showing the clear loss of choriocapillaris/RPE/EPIS/COST nasal to the fovea (arrow) and the centrifugal progression of the disease from the optic disc. Representations with an angle of 30° of SD-EDI-OCT of the macula **(A),** FAF **(B),** and FA/ICGA **(C)**

On EDI-OCT, the lesions resembled geographic atrophy with RPE and choroidal thinning and loss of the ellipsoid portion of the inner segments (EPIS), and cones outer segment tips (COST) lines. Furthermore, atrophy of the choriocapillaris was observed in 100% (22/22) of eyes and atrophy of the choriocapillaris and RPE in 91% (20/22 eyes). Additional loss of the inner retinal layers was seen in more advanced stages of atrophy in 12/22 eyes (55%) with increased visibility of the choroid, which was isoreflective in all patients (Fig. 1a).

In FAF, the lesions were hypoautofluorescent at all stages in 100% of eyes (Fig. 1b). In FA, the lesions were mostly hypofluorescent in the early phase with well-defined hyperfluorescent margins with no leakage nor blurry margins, whereas they remained hypofluorescent in ICGA, providing a better discernment of the true extent of the disease as shown in Fig. 1c.

### OCTA

**Fig. 2:**
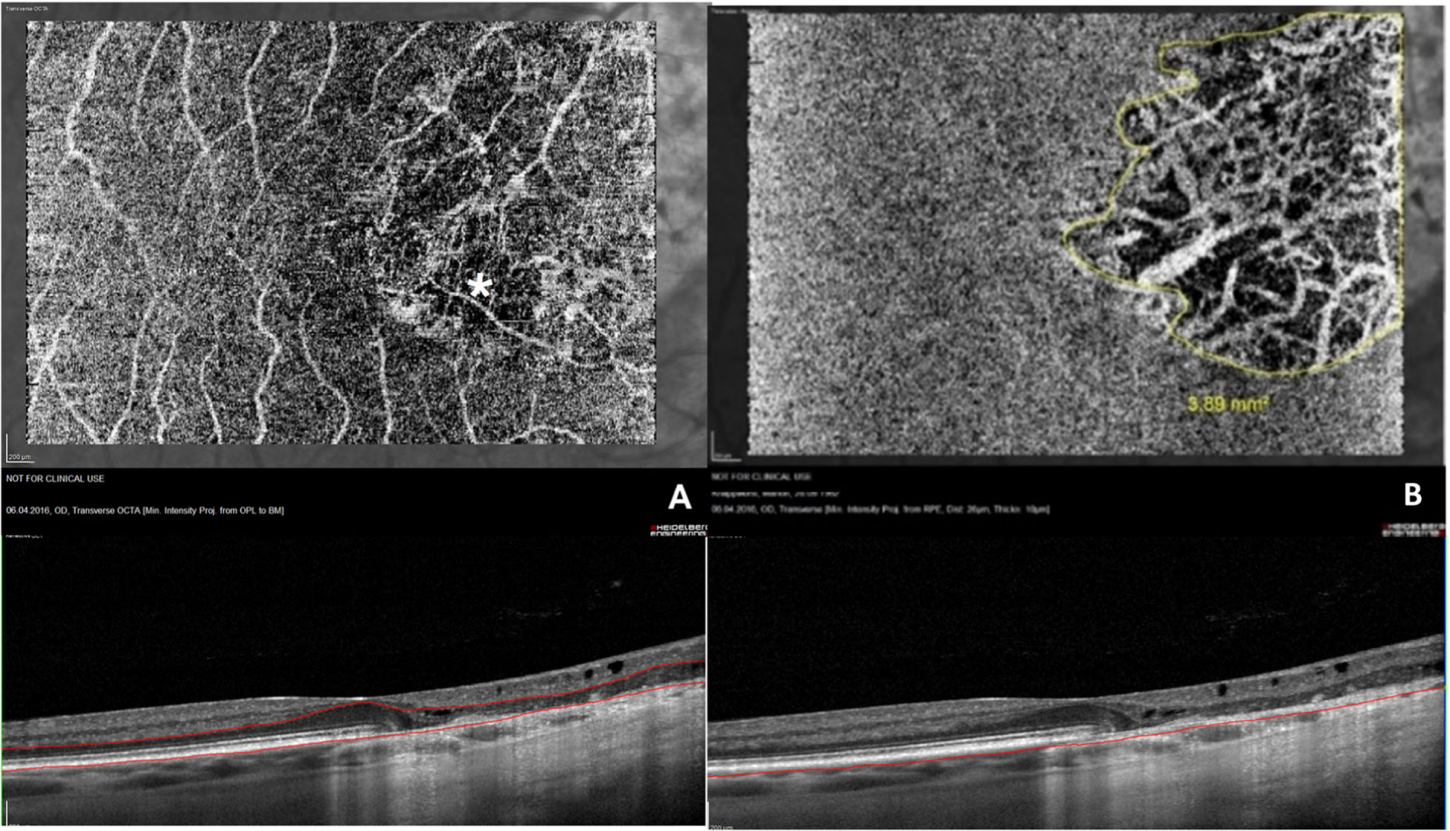
Choroidal changes in SC. Segmentation of the retina showing a defect (asterisk) of the RPE and choriocapillaris (**a**), and just the choriocapillaris in greater detail (**b**). The OCT angiograms are projections of the retina between the automatically segmented outer plexiform layer and the manually corrected basal membrane in (**a**) and at an imaginary line running 10 μm below and parallel to the manually segmented retinal pigment epithelium in (**b**), shown in the corresponding OCT B-Scans below

**Fig. 3:**
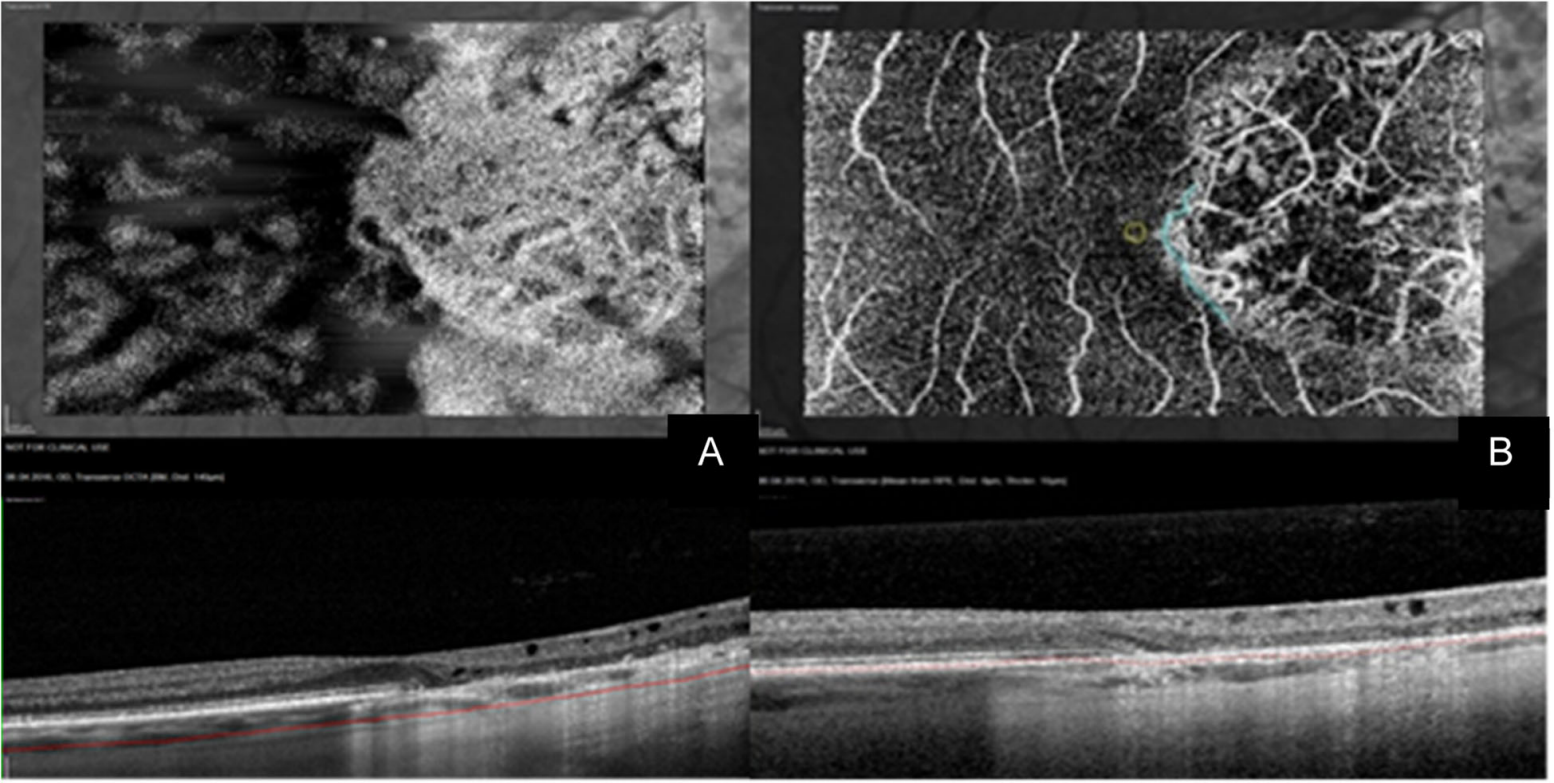
Choroidal changes in SC (continued). The hypoperfusion seen in Fig. 2 leads to a window defect responsible for the “White on Black effect” in the Haller’s Layer seen here at the top of (**a**). **b** displays the same defect at the level of the retinal pigment epithelium, showing that the area corresponding to the foveola was spared. The OCT angiograms are projections of the retina at an imaginary line running 140 μm inferiorly and parallel to the basal membrane in (**a**) and through the manually segmented retinal pigment epithelium in (**b**), shown in the corresponding OCT B-Scans below

Standardized representation of the different retinal and choroidal layers using OCTA showed that the SCP was identified correctly in 19 of 22 eyes (86%), while Sattler’s and Haller’s layer could only be identified correctly in 10 eyes (45%). DCP and RPE could be discerned in 15 eyes (68%), and choriocapillaris in 14 eyes (64%) (mean of interobserver agreement measurements: k = 0.891; range: 0.891–1; p < 0.001) (Figs. 2 and 3).

When comparing the lesions in a multimodal imaging approach, the extent of the lesions remained constant throughout FA/ICGA/FAF/EDI-OCT/OCT, with more details of the deeper layers being offered by OCTA.

The mean area of focal flow impairment and atrophy was, respectively, 2.64 mm^2^ (SD = 3.37 mm^2^) in the choriocapillaris and 2.36 mm^2^ (SD = 3.33 mm^2^) in the RPE. This showed no statistically significant difference (p = 0.39) and a high degree of inter observer agreement (Lin’s concordance correlation coefficient = 0.867; CI: 0.71–0.94). The area of choriocapillaris hypoflow was only smaller than the area of atrophy in the RPE in one eye (9.37 mm^2^ to 10.32 mm^2^, respectively).

The atrophy caused by SC led to the presence of white-on-black effect in the Haller’s layer, this referring to a large white area on a black background corresponding grossly to the atrophic area. This is shown in Fig. 3a. There were no observable differences between the 3 forms of SC.

Both figures (Fig. 3a and b) pertain to the same patient, clearly showing that the foveola was spared when viewed on the OCT of Fig. 3a and on the OCTA of Fig. 3b, which is the reason for a BCVA of 20/20 [0.1 logMAR] on examination, even though the vision had been 6/38 [0.8 logMAR] during a recent active inflammatory episode, which was successfully treated with Interferon-α.

OCTA images of 10 eyes (46%) showed significant SE. Mean BCVA of eyes with SE’s was 20/400 [1.3 logMAR] in contrast to the patients’ group without SE’s with a BCVA of 20/34 [0.23 logMAR] (Mann-Whitney U Test: z = − 2.15, p = 0.031). SE’s were only seen in 3 eyes (14%) with a BCVA better than 20/100 [0.7 logMAR]. A statistically significant “correlation”/relationship was established between low BCVA and increased presence of SE’s using a one-way ANOVA (η = 0.57, F = 9.76, p = 0.005).

## Discussion

OCTA is a non-invasive diagnostic method of assessing the retinal and choroidal vascular layers. It is currently suggested that the corollary atrophy of the choriocapillaris resulting from its hypoperfusion, as first reported as a loss of choriocapillary homogeneity on OCTA by Khan et al., causes the consecutive destruction of RPE and photoreceptors which leads to vision loss [21, 23].

In our study, OCTA images showed a clear hypoperfusion of the choriocapillaris in 100% of our patients, which compared well to the atrophic areas observed on the EDI-OCT [22].

Equally, the atrophic areas in the RPE represented by hypoperfusion in the OCTA, greatly and reliably corresponded to the same atrophic areas seen in FA, ICGA and FAF. However, the extent of choriocapillaris hypoperfusion (mean area = 2.64 mm^2^) was greater than the atrophic areas of the RPE (mean area = and 2.36 mm^2^) in all but one eye, which could be explained by the choroidal origin of the disease, even though this difference was not statistically significant. These same areas were hypoautofluorescent in all eyes, since the disease was inactive. According to the literature, hypoautofluorescent lesions with hyperautofluorescent borders were to be expected in active disease [21–23]. The study of Montorio et al. found a complete loss of detectable flow in 2 active eyes with SC, whereas choroidal vessel rarefaction and loss of choriocapillaris could be seen in inactive eyes (20 eyes) [31].

The pathophysiology of the 3 different forms of SC remains unclear. No differences could be seen between these forms in all imaging modalities and none have been described to date.

Although OCTA proved to be useful for imaging of the choriocapillaris, the representation of the deeper choroidal layers contained challenges. The Sattler’s layer examination did not reveal much information, whereas a conversion from a “black-on-white” effect to a “white-on-black” effect could be seen in the Haller’s layer below the atrophic regions, as demonstrated in the literature. This effect remains to be explained [19]. Current theories explain it as a consequence of the physics of wave propagation through the different retinal and choroidal layers, in that the propagation through the atrophic lesions is not hampered by so many layers/cells as it is in the neighbor regions, causing this “white-on-black” effect much later if at all. Another hypothesis is that this effect can be a consequence of the absence of or different flow (e.g. too slow or too fast) or characteristics of the vessels and surrounding tissues within these regions due to inflammation as first proposed by Montorio et al. [31].

The standard adjustments of the retinal and choroidal layers did not correspond very well with the structures of deeper layers.

SE’s were the main problem in patients with macular edema or scarring so that the interpretation of these images was difficult and led to the exclusion of one eye. Statistically significant differences of mean BCVA could be seen between patients with and without SE’s which may be due to a missing fixation of the patients and a longer examination time of OCTA. The newer software with faster examination time and auto-fixation program overcomes some of these problems.

One last disadvantage concerned the lack of detection of fluid dynamics (pooling, staining, leakage) even though one could argue that this provided in-depth-selective images, free of masking affects.

Although this study’s conclusions are limited by the numbers of eyes evaluated and there is missing knowledge of the pathophysiology of SC, lesions presenting in the disease can be visualized using OCTA as reliably as through the currently established techniques (ICGA, FA and FAF). The use of OCTA offers a non-invasive and high-resolution means to assess the disease in three dimensions, allowing for the ability to precisely locate the atrophic area. Furthermore, OCTA may help determine if the disease is in an active phase via, for example, identification of CNV (a known complication of this and other entities) even when ICGA is not able to do so, as reported by Mandadi et al. and as observed in tuberculous serpiginous-like Choroiditis. In this study, it was not possible to prove this hypothesis as all enrolled patients were in inactive disease stages [32].

Additionally, analogies could be seen between the changes of the choriocapillaris and choroid caused by SC and geographic atrophy. These included a focal flow impairment and rarefaction in the choriocapillaris beneath the area of the atrophic lesions, which is argued to represent non-perfused or hypo-perfused choroidal vessels with non-detectable flow in the literature [33–35].

OCTA is clinically valuable in the management of patients with SC. Even though the representation of the lesions with OCTA corresponded to the ones seen in the FA/ICGA/FAF/EDI-OCT, the inclusion of this tool in a multimodal approach for the diagnosis and management of SC should be considered as an extra or alternative examination.

## Conclusions

OCTA is able to assess vascular lesions of the choroid and retina in patients with serpiginous Choroiditis with a high degree of correlation to other diagnostic modalities.

## Data Availability

Data and materials related to this work are available from the corresponding author upon reasonable request.

## Abbreviations

BCVA: Best corrected visual acuity
BM: Basement membrane
CNV: Choroidal neovascularisation
COST: Cones outer segment tips
DCP: Deep capillary plexus
EDI-OCT: Enhanced depth imaging optical coherence tomography
EPIS: Ellipsoid portion of the inner segments
FA: Fluorescein angiography
FAF: Fundusautofluorescence
ICGA: Indocyanine green Angiography
ILM: Inner limiting membrane
OCTA: Optical coherence tomography angiography
RPE: Retinal pigment epithelium
SC: Serpiginous choroiditis
SCP: Superficial capillary plexus
SD: Standard deviation
SE: Segmentation error
